# Automated and real-time structure solution using 3D electron diffraction

**DOI:** 10.1107/S1600576725008404

**Published:** 2025-10-24

**Authors:** Yi Luo, Yuwei Deng, Bin Wang, Junshu Chen, Weimin Yang, Xiaodong Zou

**Affiliations:** a State Key Laboratory of Green Chemical Engineering and Industrial Catalysis, Sinopec Shanghai Research Institute of Petrochemical Technology, 1658 Pudong Beilu, Shanghai, 201208, People’s Republic of China; b Department of Chemistry Stockholm University Stockholm SE-106 91 Sweden; c Wallenberg Initiative Materials Science for Sustainability Stockholm University Stockholm SE-106 91 Sweden; SLAC National Accelerator Laboratory, Menlo Park, USA

**Keywords:** 3D electron diffraction, automated structure solution, *Instamatic-solve*

## Abstract

Three-dimensional electron diffraction (3D ED) has emerged as a powerful tool for solving the structures of small crystals down to nanometre-scale sizes. Despite advancements in automating data acquisition for 3D ED, the subsequent data processing and structure solution have largely relied on human intervention and have been mostly conducted offline. This reliance on expertise in electron crystallography and the lack of real-time feedback on data quality and structural information have limited the broader adoption of 3D ED. Here, we introduce *Instamatic-solve*, a fully automated, real-time structure solution pipeline for 3D ED deployed on a JEOL JEM 2100 transmission electron microscope. *Instamatic-solve* streamlines the entire process by automating the subsequent data processing and structure solution, providing real-time assessments of data quality and structural information. Moreover, the pipeline can handle offline 3D ED data acquired from various transmission electron microscope platforms. Using *Instamatic-solve*, we have successfully solved the crystal structures of diverse materials, including seven inorganic zeolites, two inorganic–organic hybrids and four organic molecules (including pharmaceuticals), all within 2 min. *Instamatic-solve* mimics the typical manual structure solution process, and its outcomes depend heavily on data quality. Our results indicate that a routine and reliable structure solution is achievable in most cases, provided that the data meet critical quality criteria, namely completeness ≥50% and resolution better than 1.0 Å. By enabling efficient, automated and real-time structure solution for crystalline materials, *Instamatic-solve* spans various scientific disciplines.

## Abstract 2

A fully automated, real-time structure solution pipeline, *Instamatic-solve*, has been developed by linking *XDS* and *SHELXT* to the continuous-rotation electron diffraction method within *Instamatic*. *Instamatic-solve* automatically handles data processing and structure solution right after data collection, providing real-time assessments of data quality and structural information for efficient analysis.

## 1. Introduction

For over a century, single-crystal X-ray diffraction (SCXRD) has been the primary technique for determining the structures of crystalline materials (Thomas, 2012 ▸; Gerstner, 2011 ▸). Modern SCXRD structure solution is routine and straightforward, aided by commercial and open-source automated real-time structure solution pipelines (Holton & Alber, 2004 ▸; Matsumoto *et al.*, 2021 ▸; Viswanathan *et al.*, 2019 ▸; Rigaku, 2020 ▸; Bruker, 2016 ▸). However, SCXRD requires sufficiently large crystals (>5 × 5 × 5 µm) to harvest adequate diffraction signals (McCusker & Baerlocher, 2013 ▸; Yang *et al.*, 2022 ▸). For smaller, submicrometre-sized crystals, powder X-ray diffraction (PXRD), which measures diffraction from millions of crystals at once, is often used. Yet, the resulting peak overlaps can complicate structure solution, especially for those structures with low symmetries and/or large unit cells (McCusker & Baerlocher, 2013 ▸; Yang *et al.*, 2022 ▸; Li & Sun, 2017 ▸; Zhou *et al.*, 2016 ▸).

By contrast, the stronger interaction between electrons and matter makes electron diffraction well suited for structural studies of crystals that are too small for SCXRD (Gemmi *et al.*, 2019 ▸). Three-dimensional electron diffraction (3D ED), which is conceptually similar to SCXRD, has been extensively used for structural studies in the past two decades. Several automated or semi-automated data collection protocols including automated diffraction tomography (Kolb *et al.*, 2007 ▸), rotation electron diffraction (Zhang *et al.*, 2010 ▸; Wan *et al.*, 2013 ▸), continuous-rotation electron diffraction (cRED) (Wang, Yang *et al.*, 2018 ▸; Cichocka *et al.*, 2018 ▸), microcrystal electron diffraction (Nannenga *et al.*, 2014 ▸; Nannenga, 2020 ▸), precession electron diffraction tomography (Gemmi *et al.*, 2013 ▸; Kolb *et al.*, 2019 ▸), serial electron diffraction (SerialED) (Smeets, 2018 ▸; Bücker *et al.*, 2020 ▸; Hogan-Lamarre *et al.*, 2024 ▸) and serial rotation electron diffraction (SerialRED) (Wang *et al.*, 2019 ▸; Luo *et al.*, 2023 ▸) have been developed for 3D ED. These methods have enabled successful structure determination of a wide range of polycrystalline materials [*e.g.* zeolites, metal–organic frameworks (MOFs) and drug molecules] which prove challenging for SCXRD and/or PXRD (Gemmi *et al.*, 2019 ▸; Huang, Grape *et al.*, 2021 ▸; Palatinus *et al.*, 2017 ▸; Luo *et al.*, 2017 ▸; Xu *et al.*, 2019 ▸; Yonekura *et al.*, 2015 ▸).

3D ED structural studies include the steps of (1) crystal screening, (2) data acquisition, (3) data processing and (4) structure solution, all of which require effort and expertise (Li & Sun, 2017 ▸; Gruene & Mugnaioli, 2021 ▸; Huang, Willhammar & Zou, 2021 ▸; Saha *et al.*, 2022 ▸; Yonekura *et al.*, 2023 ▸; Bengtsson *et al.*, 2022 ▸). While efforts have been made to automate these steps and streamline the workflow to rival SCXRD in convenience and routine, current advancements primarily address crystal screening and data acquisition (Winter, 2010 ▸; Vonrhein *et al.*, 2011 ▸). For instance, *Instamatic*, an open-source Python package, integrates the SerialRED and SerialED methods for automated crystal screening and electron diffraction data collection (Smeets, 2018 ▸; Wang *et al.*, 2019 ▸; Luo *et al.*, 2023 ▸). While real-time data processing is available for SerialRED, it has mainly been used for unit-cell determination. Further data analysis and dataset merging still rely on offline, stepwise and command-line-based operations. Consequently, data processing and structure solution in 3D ED remain largely manual and time consuming. Moreover, the absence of real-time automation tools for seamless 3D ED data processing and structure solution after data collection can lead to redundant or insufficient datasets, poor data quality and insufficient data completeness. These pose challenges for effective data collection and structural analysis using 3D ED and hamper its widespread adoption (Wang *et al.*, 2019 ▸; Luo *et al.*, 2023 ▸; Ito *et al.*, 2021 ▸, Bengtsson *et al.*, 2022 ▸).

*Autochem* is the first reported pipeline to integrate all four steps for a real-time and automated 3D ED structure solution. However, it relies on the commercial *CrysAlis Pro* software (Rigaku, 2020 ▸) and is currently limited to the XtaLAB Synergy-ED diffractometer. An open-source pipeline, *AutoMicroED*, has been proposed for semi-automated processing of offline 3D ED data, specifically for small-molecule structure solution (Powell *et al.*, 2021 ▸). However, there is a strong need for a more accessible 3D ED pipeline that is capable of fully automated real-time data processing and structure solution.

To this end, here we introduce the structure solution pipeline *Instamatic-solve*. *Instamatic-solve* is built by interfacing calls to the *XDS* and *SHELXT* programs within *Instamatic* (Smeets *et al.*, 2018 ▸; Sheldrick, 2015 ▸; Kabsch, 2010 ▸; Smeets, 2018 ▸). We validated its automated real-time structure solution performance using two zeolites and tested its automated offline structure solution capabilities on five zeolites, two inorganic–organic hybrids and four small molecules. The offline 3D ED data of these samples were collected on either JEOL or Thermo Fisher Scientific (TFS) transmission electron microscopes (TEMs) equipped with different detectors (ASI Timepix, Ceta D, OneView and TemCam XF416) (Gallagher-Jones *et al.*, 2020 ▸; Luo, Clabbers *et al.*, 2022 ▸; Gorelik *et al.*, 2023 ▸). Using *Instamatic-solve*, the crystal structures of all these tested samples have been successfully solved within 2 min. Our results demonstrate that *Instamatic-solve* can offer correct structure solutions for data with high enough completeness (≥50%) and high resolution (better than 1.0 Å). When the data completeness is high (≥90%), the requirement on resolution can be reduced to 1.2 Å, which is in line with the requirements on data quality for SCXRD structure solution (Sheldrick, 1990 ▸). *Instamatic-solve* enables effective data processing and structure solution without any human interactions, facilitating the application of the technique by novices.

## 2. Results and discussion

### 2.1. Proposed automated structure solution pipeline *Instamatic-solve*

**Figure 1:**
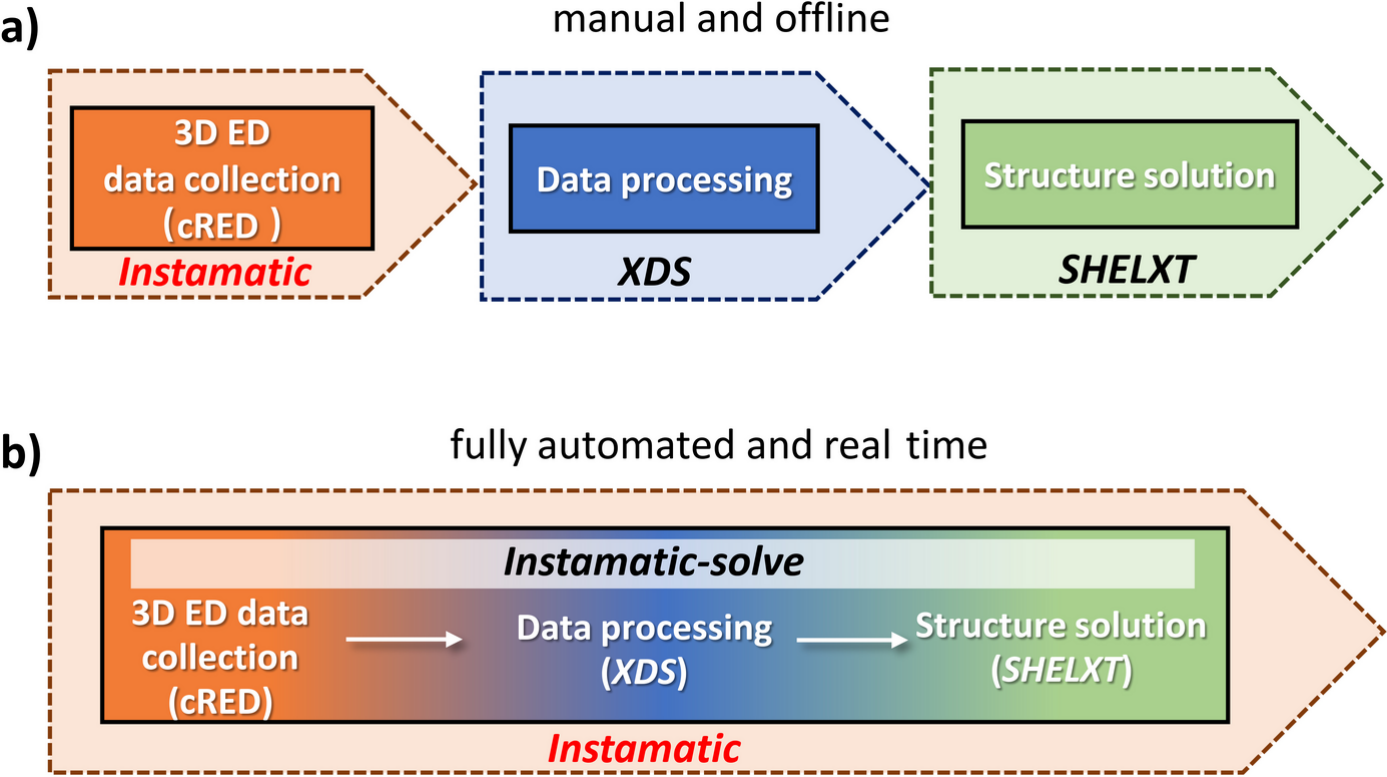
Flowcharts illustrating the procedures of (*a*) a typical manual and offline structure solution process and (*b*) our proposed fully automated and real-time *Instamatic-solve* pipeline (implemented in *Instamatic* for the cRED method) for 3D ED.

In the well established offline, stepwise and manual 3D ED structure solution workflow, *XDS* (Kabsch, 2010 ▸) is commonly used for data processing, while *SHELXT* (Sheldrick, 2015 ▸) is employed for structure solution, both requiring user expertise in crystallography [Fig. 1 ▸(*a*)]. In the previous development of SerialRED (Wang *et al.*, 2019 ▸; Luo *et al.*, 2023 ▸), *XDS* was linked to SerialRED within *Instamatic* for real-time data processing. However, the data completeness of most single 3D ED datasets obtained from SerialRED was insufficient for direct structure solution using *SHELXT*. In this context, real-time data processing primarily served for unit-cell determination, while further data analysis and dataset merging were required. To facilitate this, *edtools* was used to call *XDS* for offline, semi-automated data processing and phase analysis, which still required manual command-line input. Structure solution was then performed offline using *SHELXT*. To streamline the entire process to perform fully automated and real-time structure solution, we developed *Instamatic-solve*, by linking *XDS* and *SHELXT* to the cRED method, which is implemented in *Instamatic* for acquiring high-quality single 3D ED datasets [Fig. 1 ▸(*b*)]. *Instamatic-solve* is embedded within and largely based on *Instamatic*, and it also supports automated structure solution using offline 3D ED datasets collected from diverse transmission electron microscope setups.

**Figure 2:**
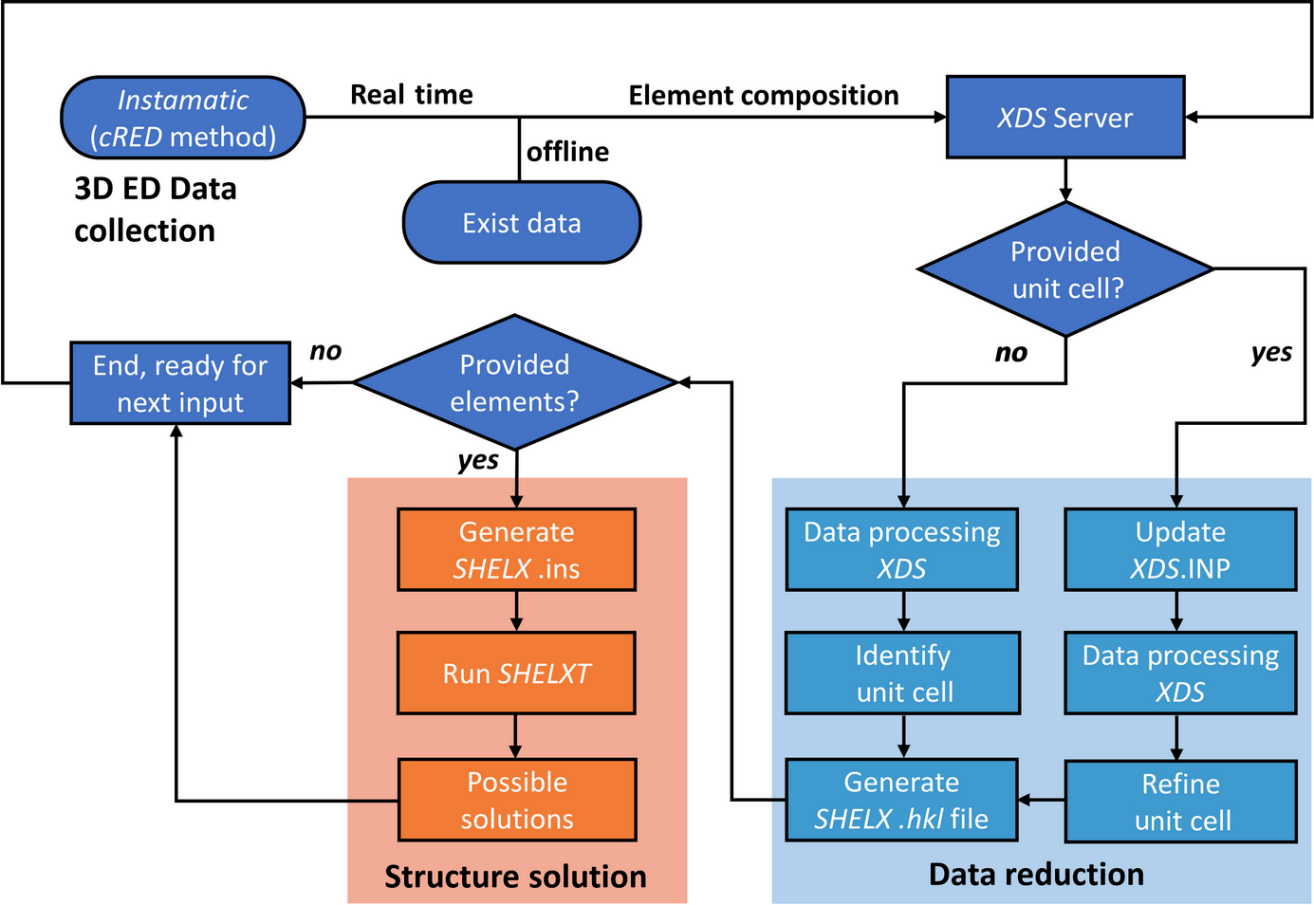
Flowchart depicting the real-time and offline automated structure solution pipeline *Instamatic-solve* for 3D ED. The automated data reduction steps are highlighted in light blue, and the structure solution steps are in orange.

For real-time automated structure solution, *Instamatic-solve* was deployed on a JEOL JEM 2100 TEM equipped with an ASI Timepix detector. For offline use, *Instamatic-solve* can be run on any computer running Windows 7 or later, provided the 3D ED data (in SMV, CBF, RAXIS, TIFF or other *XDS*-compatible formats) are available. Before each pipeline run, users must supply a preliminary chemical composition for *SHELXT*, while input of the space group and unit-cell parameters is optional (Fig. 2 ▸). The GUI of *Instamatic* with a provided chemical composition is shown in Fig. S1 (in the supporting information). During pipeline execution, the cRED method implemented in *Instamatic* is responsible for 3D ED data collection. It also automatically records all necessary experimental parameters and generates the required *XDS.inp* and SMV files without any user intervention for the real-time automated structure solution (Wang, Yang *et al.*, 2018 ▸). For the offline automated workflow, users only need to provide a folder path of the existing 3D ED data files. Once the data are ready, either real time or offline, *XDS* automatically processes the data, determines or refines the unit-cell parameters (run only once), identifies the Laue group, and generates a standard *SHELX.hkl* intensity file. For the resolution cutoff, the default resolution range is defined from 20 to 0.8 Å in the *XDS.inp* file. Therefore, all datasets will initially be processed with a cutoff at 0.8 Å. Depending on the statistics for this resolution range shown in *CORRECT.Lp*, *Instamatic-solve* will adjust the resolution cutoff when generating the *SHELX.hkl* intensity files using *XDSCONV.inp*. If reflections at 0.8 Å do not meet the criteria of *I*/σ(*I*) ≥ 0.3 and CC_1/2_ is larger than or equal to 0.5 and flagged with a star, the cutoff will be automatically reduced to the highest resolution that satisfies these criteria. *Instamatic* in the end generates *SHELX.ins*, enabling *SHELXT* to extract the required information and perform the structure solution, including atom-type and space-group assignments.

Notably, *Instamatic-solve* mimics a typical manual workflow, using *XDS* for data processing and *SHELXT* for structure solution. As in SCXRD, the success of an automated 3D ED structure solution depends heavily on data quality and on the capabilities of *XDS* and *SHELXT*. For instance, if the data completeness is below 50%, *XDS* may incorrectly identify the unit-cell parameters or space groups. Likewise, if the resolution is lower than 1.2 Å, *SHELXT* may fail to deliver viable structure solutions (Sheldrick, 1990 ▸). Furthermore, for crystals with complex compositions, particularly those containing multiple elements or elements with similar atomic numbers, *SHELXT* may encounter challenges in assigning the correct atom types.

### 2.2. The reliability of *Instamatic-solve*

**Figure 3:**
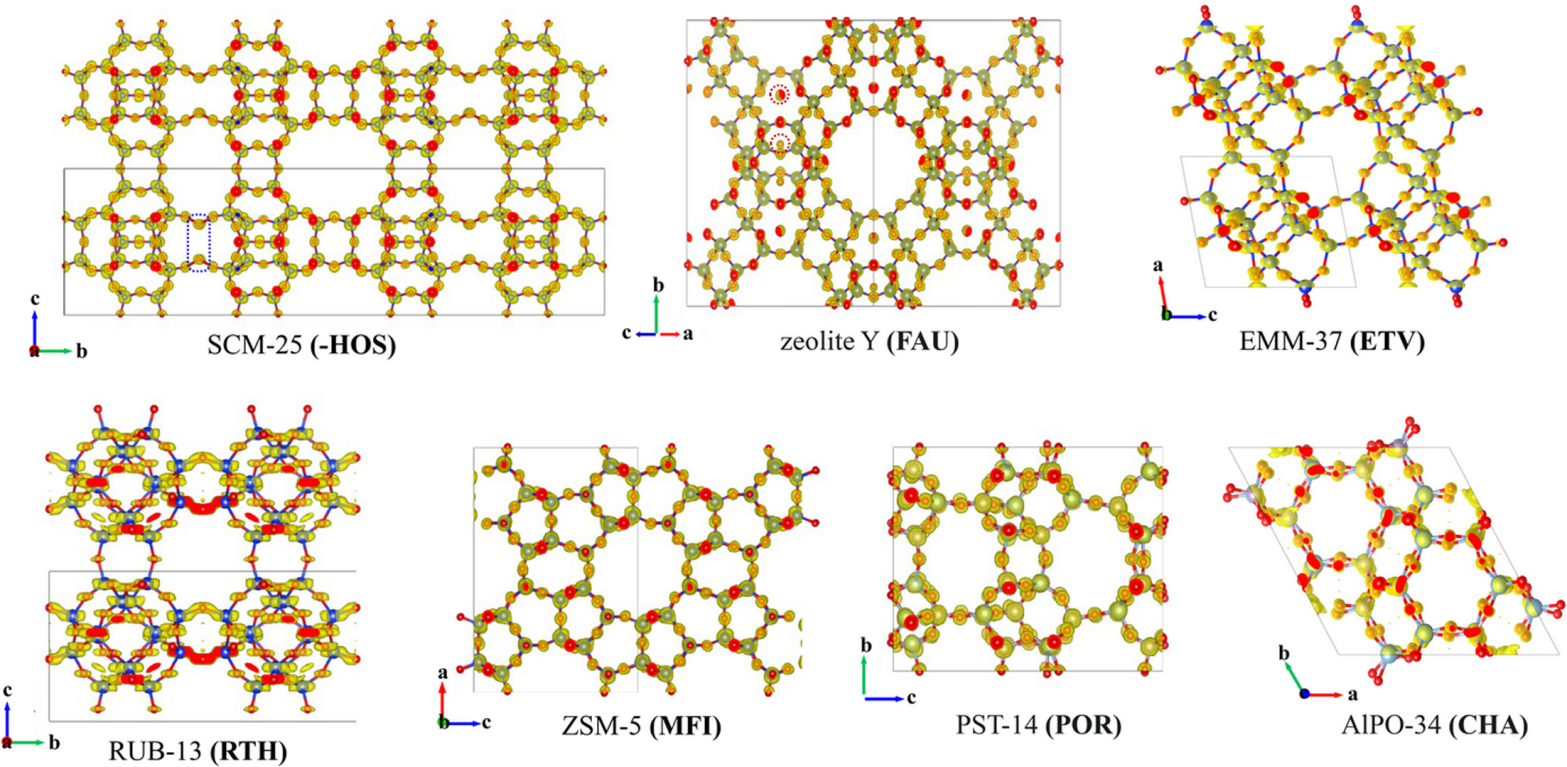
Observed electrostatic potential maps and corresponding framework structures of different zeolites solved using *Instamatic-solve*. Electrostatic potential maps were generated from the structure-factor data (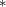
*.fcf*) using the Fourier synthesis function in *VESTA*, revealing the 3D distribution of the electrostatic potential within the structure. SCM-25 (-**HOS**), zeolite Y (**FAU**), EMM-37 (**ETV**), RUB-13 (**RTH**), ZSM-5 (**MFI**), PST-14 (**POR**) and AlPO-34 (**CHA**) crystallize in orthorhombic, cubic, triclinic, monoclinic, orthorhombic, tetragonal and hexagonal systems, respectively. The electrostatic potential map for **RTH** is less well resolved, mainly due to the low data completeness (47.7%). The blue dashed frame highlights the disordered T atoms and missing O atoms in the SCM-25 framework. The red dashed frame indicates the positions of extra-framework water molecules and/or Na^+^ cations in **FAU**.

For real-time automated structure solution, *Instamatic-solve* was evaluated using two zeolites of known structure, SCM-25 (framework type -**HOS**) and faujasite (framework type **FAU**). SCM-25 is a germanosilicate, whereas **FAU** is an aluminosilicate. The real-time *Instamatic-solve* workflow is shown in Videos 1 and 2 in the supporting information. Because SCM-25 contains mixed Ge/Si sites and **FAU** contains mixed Al/Si sites, *SHELXT* cannot directly handle the mixed tetrahedral (T) sites. Both zeolites therefore were treated as pure silica ([SiO_2_]_*n*_), using [Si1O2] as the element composition for structure solution. SCM-25 was successfully solved in 5.0 min, encompassing data acquisition, data processing and structure solution. This time consumption is much shorter than the time typically required by standard SCXRD experiments (Bloch *et al.*, 2015 ▸). The crystallographic data and structure solution results for SCM-25 are listed in Table 1 ▸, and the obtained framework is shown in Fig. 3 ▸. The 3D ED dataset exhibited 89.4% completeness (resolution 0.8 Å), and *XDS* automatically identified a *C*-centered orthorhombic cell (*a* = 14.65, *b* = 51.87, *c* = 13.10 Å). Solutions of the structures (*SHELX .res* files) with the same topology but different symmetries or settings (*Cmmm*, *Cmm*2, *Amm*2 and *C*222) were obtained. In the highest symmetry *Cmmm*, all ten framework T sites (including one disordered) and 24 framework O atoms in the asymmetric unit were directly located. Two O atoms associated with the disordered T site [defects, T(OT)_3_OH species] were missing, and their positions can be inferred from established zeolite chemistry principles (Flanigen *et al.*, 1991 ▸). These real-time structure solution results agree with earlier, manually obtained ones (Luo, Fu *et al.*, 2022 ▸). For **FAU**, with data completeness of 99.2% and resolution of 0.8 Å, a cubic cell with *a* = 25.08 Å and space group *Fd*3*m* was identified by *SHELXT*. The complete framework structure, including one T atom and four O atoms in the asymmetric unit, was readily solved, and extra-framework species (water molecules and/or Na^+^ cations) were also located (Fig. 3 ▸).

To run the offline automated structure solution, the path to the folder containing the existing 3D ED data (SMV format) and the corresponding *XDS.inp* file should be provided to *Instamatic*. Its performance was assessed on the structure solution of five known zeolite structures with different crystal systems. These zeolites are of **ETV**, **RTH**, **MFI**, **POR** and **CHA** framework types, and their crystal systems are triclinic (*P*1), monoclinic (*C*2/*m*), orthorhombic (*Pnma*), tetragonal (*P*42_1_*c*) and hexagonal (*R*3*m*), respectively (Kapaca *et al.*, 2019 ▸; Seo *et al.*, 2018 ▸; Baerlocher & McCusker, 2019 ▸). For *Instamatic-solve* input, **ETV**, **RTH** and **MFI** were treated as pure silica ([SiO_2_]_*n*_), whereas **CHA** and **POR** were considered aluminophosphates ([AlPO_4_]_*n*_). A demonstration of the offline automated structure solution is provided in Video 3 in the supporting information. The offline 3D ED datasets of these zeolites were all collected via the cRED method implemented in *Instamatic* and had data completeness of 55.8, 47.7, 87.2, 94.7 and 60.3%, respectively (at 0.8 Å resolution; Table 1 ▸). Despite some datasets approaching a low 50% completeness, all five framework structures were successfully solved within 1 min (Fig. 3 ▸ and Table 1 ▸). The space groups assigned by *SHELXT* for **ETV**, **MFI** and **POR** are consistent with the reported ones, while for **RTH** and **CHA**, subgroups (*Cm* and *R*3) were assigned instead of the previously published *C*2*/m* and *R*3*m*. Note that the symmetry of a zeolite framework may vary depending on the synthesis conditions and/or the presence of guest species (*e.g.* H_2_O) within the pores (Hogan-Lamarre *et al.*, 2024 ▸). The **RTH** and **CHA** samples used in this study may possess lower symmetries than previously reported, or their symmetries may have been reduced due to the removal of guest species under vacuum conditions in the TEM.

These results of the real-time and offline automated structure solution underscore the reliability and efficiency of *Instamatic-solve* for zeolite structures with varying data quality. The underlying principles of real-time and offline automated structure solution are comparable; their main distinction lies in their respective advantages and use cases. Real-time solutions provide immediate feedback on data quality and structural features during a data collection session, while offline solutions enable rapid structure determination once datasets are ready. Together, they offer a versatile platform for diverse research needs while minimizing manual intervention.

### 2.3. The capability and adaptability of *Instamatic-solve*

**Figure 4:**
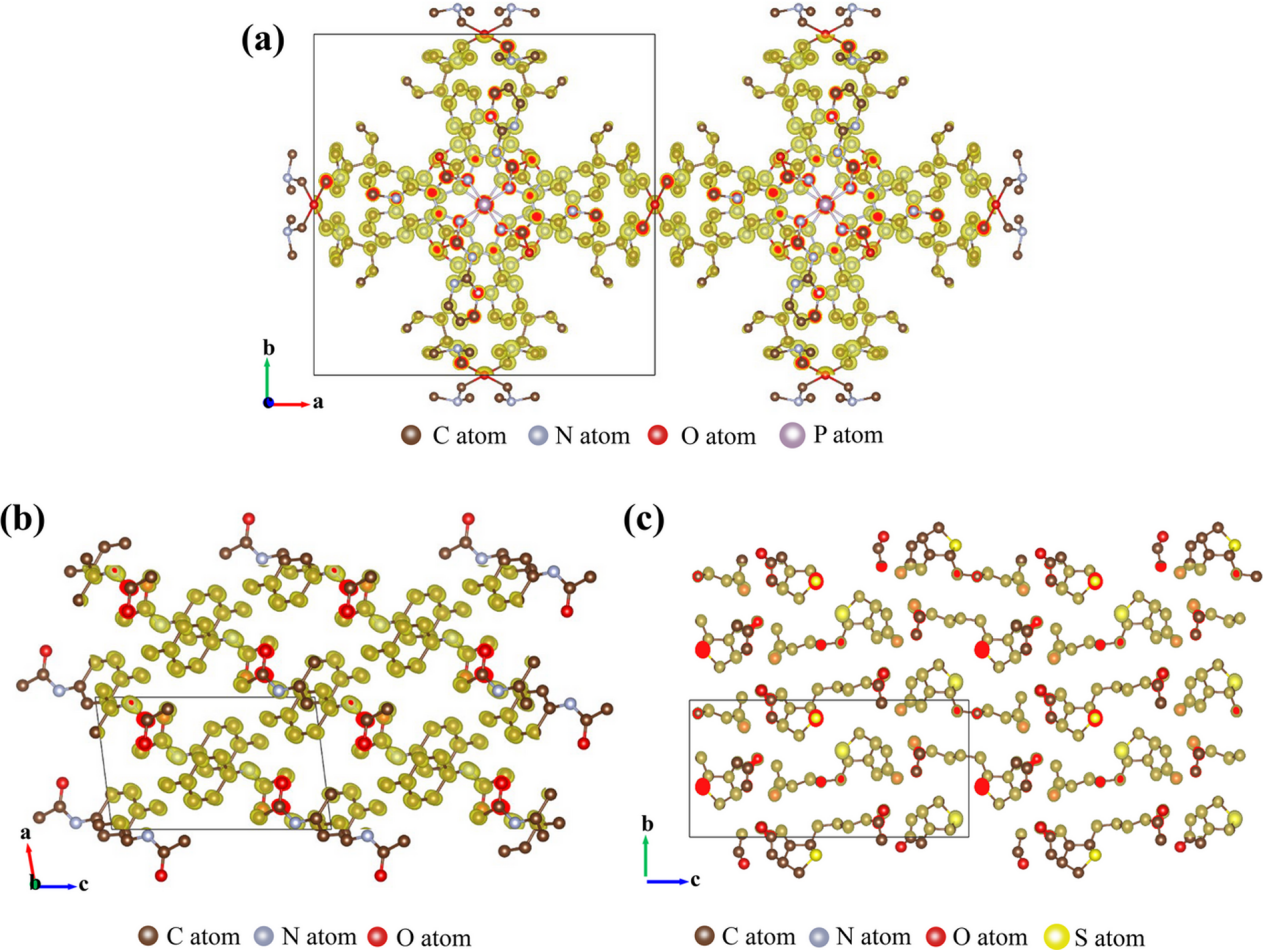
Observed electrostatic potential maps and corresponding solved structures of (*a*) CAU-36, (*b*) acetamino­phen and (*c*) biotin.

Encouraged by the successful automated structure solution results of inorganic zeolites, we further evaluated the capability of *Instamatic-solve* for the structure solution of an inorganic–organic hybrid MOF material CAU-36 and two organic small molecules (biotin and acetamino­phen) (Table 2 ▸). Their offline 3D ED datasets were all collected using the cRED method implemented in *Instamatic*. CAU-36 has a complex element composition of [Co1Ni1P1C1N1O1] (Wang, Rhauderwiek *et al.*, 2018 ▸) and its data completeness is 94.9% (resolution 0.8 Å). *SHELXT* identified a tetragonal cell (*a* = *b* = 21.95, *c* = 8.74 Å) in space group *P*4*c*2. The complete framework structure, comprising 23 symmetry-independent atoms, was directly solved [Fig. 4 ▸(*a*), Video 4 in the supporting information]. However, due to the similar atomic numbers of Co and Ni, and of C, N and O, *SHELXT* failed to assign these atom types correctly in the structure. Nonetheless, the atom types can be assigned correctly and straightforwardly on the basis of the chemical information of the building blocks used in the synthesis. For acetamino­phen and biotin, *Instamatic-solve* was run with [C1N1O1] and [C1N1O1S1] as the respective inputs. With data completeness of around 90% (resolution 0.8 Å), all non-hydrogen atoms were located [Figs. 4 ▸(*b*) and 4 ▸(*c*)]. Similarly to the case of CAU-36, *SHELXT* could not distinguish between C, N and O atoms in either acetamino­phen or biotin, yet these can be unambiguously assigned by referring to their known molecular structures and the typical bond distances of C=O (1.20 Å), C—N (1.48 Å) and C—C (1.54 Å) (Welberry, 2021 ▸).

The 3D ED datasets for the above zeolites, MOF and small molecules were all collected on a JEOL JEM 2100 TEM equipped with an ASI Timepix detector. In a TEM configuration for 3D ED data acquisition, the characteristics of the detector, such as its electron detection sensitivity, background levels, dynamical range and read-out speed, can affect the quality of the data and ultimately the results of the structure solution. To explore the adaptability of *Instamatic-solve* to other instrumentation, we tested additional offline 3D ED datasets obtained from different research groups using various TEM platforms. Specifically, SCM-34 (Luo, Clabbers *et al.*, 2022 ▸) data were collected on a TFS Themis Z (OneView camera), PPEA {9,10-bis-[(perchlorophenyl)ethynyl]anthracene} (Gorelik *et al.*, 2023 ▸) on a Glacios Cryo-TEM (Ceta-D camera) and AVAAGA peptide (Gallagher-Jones *et al.*, 2020 ▸) on a Tecnai F30 (TemCam XF416 camera). By converting the raw data to SMV format and updating key parameters (detector distance, physical size of pixels, wavelength, rotation axis *etc*.) in the *XDS.inp* file, all structures have been successfully solved using *Instamatic-solve*.

SCM-34 is a hybrid aluminophosphate (|(C_6_N_3_H_13_)_2_|[P_4_Al_2_O_18_H_6_]) which crystallizes in the triclinic space group *P*1 and contains 21 symmetry-independent non-hydrogen atoms in its unit cell. The structure can be solved at 0.8 Å resolution with a data completeness of 51.1%, including all non-hydrogen atoms [Fig. S2(*a*)]. PPEA (C_30_H_9_Cl_10_) has a monoclinic unit cell (space group *P*2_1_/*c*) with 20 symmetry-independent non-hydrogen atoms and a data completeness of 68.1% (resolution 0.8 Å, Table 3 ▸). In the solved structure, all C and Cl positions were correctly assigned [Fig. S2(*b*)]. The AVAAGA peptide (C_10_H_23_N_3_O_6_) possesses a large ortho­rhombic unit cell (*a* = 11.36, *b* = 4.73, *c* = 39.59 Å, *P*2_1_2_1_2_1_) (Gallagher-Jones *et al.*, 2020 ▸). *XDS* assigned a monoclinic unit cell (*a* = 11.30, *b* = 4.70, *c* = 38.86 Å, β = 90.74, *P*2) owing to a low data completeness of 47.5% (resolution 0.8 Å). *SHELXT* determined the crystal structure with the correct molecule structure in the subgroup *P*2_1_ [Fig. S2(*c*)].

These results collectively highlight the robustness and adaptability of *Instamatic-solve* across a diverse range of TEM platforms and material types, demonstrating that the pipeline can accommodate variations in detector characteristics.

### 2.4. Criteria for a successful structure solution using *Instamatic-solve*

*Instamatic-solve* has demonstrated high reliability for both real-time and offline automated structure solution, accommodating various material types and adapting to different TEM platforms. Nonetheless, its performance is critically dependent on the quality of the 3D ED data. To assess this impact, we systematically tested *Instamatic-solve* with single, unmerged 3D ED datasets from **ETV**, **RTH**, **MFI**, **FAU**, **POR**, **CHA**, acetamino­phen and biotin at different resolution and completeness cutoffs (Tables S1 and S2).

These tests highlight three key factors, data completeness, data resolution and crystal symmetry, as crucial for successful automated structure solution. As demonstrated by our results, datasets with completeness ≥50% and resolution better than 1.0 Å show a high probability of yielding correct solutions (Tables 1 ▸, 2 ▸, 3 ▸, S1 and S2). These criteria are consistent with previous studies on the influence of data resolution and completeness on 3D ED structure solution (Ge *et al.*, 2021 ▸). In lower-symmetry cases with limited data completeness, success is highly sensitive to the decrease in resolution (data completeness remains nearly constant). For example, **ETV** (*P*1 symmetry, 55.8% completeness) could not be solved when the resolution cutoff increased from 1.0 to 1.2 Å, and solution of **RTH** (*Cm* symmetry, 47.7% completeness) only succeeded at 0.8 Å. By contrast, successful solutions of higher-symmetry and/or higher-completeness cases such as **MFI** (*Pnma*, 87.2%), **FAU** (*Fd*3*m*, 99.2%), **POR** (*P*42_1_*c*, 94.7%) and **CHA** (*R*3, 60.3%) were achieved even with the resolution cutoff raised to 1.2 Å. Moreover, for datasets at 0.8 Å resolution, reducing completeness from about 90 to 50% still allowed most structures to be correctly solved (Table S2).

Within a given unit-cell volume, higher-symmetry structures generally involve fewer parameters and thus impose less stringent requirements on both resolution (Table S1) and completeness (Table S2). For instance, **POR** (*P*42_1_*c*) was correctly solved with just 40% completeness (resolution 0.8 Å) or a resolution of 1.3 Å (completeness 93.4%). Furthermore, the reduced angular coverage requirement of high-symmetry crystals allows more complete data collection within the limited rotation range of TEM stages (typically up to ∼120°). In contrast, low-symmetry systems (such as **ETV** and **RTH**) pose greater challenges in acquiring data with high completeness. An analysis of the ratio between unique reflections and parameters (*N*_reflections_/*N*_parameters_) suggests that values ≥10 typically correlate with correct structure solutions (Tables S1 and S2). The number of parameters refers to atomic coordinates (*x*, *y*, *z*) and isotropic displacement parameters (*U*_iso_) that need to be determined in structure solution. Parameters for atoms located at special positions, where certain coordinate values are fixed by symmetry (*e.g.* 0.25, 0.5, 1.0 *etc*.), were excluded from the count. These criteria also apply to small molecules such as acetamino­phen and biotin, mirroring the trends observed in inorganic zeolites.

Nowadays, the quality of 3D ED data from many sub­micrometre-sized crystals can easily meet the level required for successful automated structure solution with *Instamatic-solve*. The dual-space method employed by *SHELXT* has considerably lowered the data quality threshold while increasing structure solution reliability. Moreover, *Instamatic-solve* offers real-time assessments of data quality during a data collection session by parsing data resolution and completeness from the *CORRECT.Lp* file output by *XDS*. This immediate feedback helps users determine when sufficient data have been collected, thereby improving the efficiency of data collection and the structure solution.

The source code of *Instamatic-solve* is publicly available, allowing users to insert additional command flags for more complex dataset analyses. By mimicking manual workflows through *XDS* for data processing and *SHELXT* for structure solution, *Instamatic-solve* delivers results comparable to manual methods yet offers significantly greater convenience and speed. Although it currently does not provide automated structure refinement, the solved structures are sufficiently accurate to provide key atomic-scale insights into crystalline materials.

## 3. Code availability

*Instamatic-solve*, which is implemented in the *Instamatic* software Python package, is available from https://github.com/Junschen1/instamatic under the terms of GNU General Public License v.3.0. For detailed installation and usage instructions, please refer to the Supplementary Notes in the supporting information.

## 4. Conclusions

In summary, we present *Instamatic-solve*, a fully automated, real-time structure solution pipeline for 3D ED structure solution. By integrating the programs *XDS* and *SHELXT* with *Instamatic*, a Python package for automated electron diffraction data collection, this pipeline streamlines the entire workflow from data collection and data processing to structure solution. *Instamatic-solve* has successfully and efficiently solved the structures of a wide range of crystalline materials, including inorganic zeolites, inorganic–organic hybrids, and organic pharmaceuticals and peptides, regardless of variations in crystal symmetry and data quality. Our systematic tests indicate that *Instamatic-solve* consistently delivers correct structure solutions when 3D ED data meet quality criteria of completeness ≥50% and resolution better than 1.0 Å. For high-symmetry structures, it is inherently easier to obtain data with a high completeness and high reflection-to-parameter ratio, enabling a higher success rate of structure solution. *Instamatic-solve* can be adapted to JEOL and TFS TEM platforms with different detectors (ASI Timepix, Ceta-D, OneView and TemCam XF416). Notably, even users with limited expertise in crystallography can efficiently perform structure solution and obtain rapid structural insights. We anticipate that *Instamatic-solve* will not only advance the use of 3D ED techniques but also open up new opportunities for crystalline materials development.

## 5. Related literature

The following references are cited in the supporting information: Karplus & Diederichs (2012 ▸), Roslova *et al.* (2020 ▸), Wang *et al.* (2025 ▸).

## Supplementary Material

Video_1, preparation for automated real-time structure solution. DOI: 10.1107/S1600576725008404/te5157sup2.mp4

Video_2, automated real-time structure solution of SCM-25. DOI: 10.1107/S1600576725008404/te5157sup3.mp4

Video_3, automated offline structure solution of ETV. DOI: 10.1107/S1600576725008404/te5157sup4.mp4

Video_4, automated offline structure solution of CAU-36. DOI: 10.1107/S1600576725008404/te5157sup5.mp4

Supporting information, including the method details and results of the automated structure solution. DOI: 10.1107/S1600576725008404/te5157sup1.pdf

## Figures and Tables

**Table 1:** Summary of the crystallographic data and automated structure solution details for known zeolites The 3D ED data of all the samples were collected using the cRED method implemented in *Instamatic*.

Sample	SCM-25	Zeolite Y	EMM-37	RUB-13	ZSM-5	PST-14	AlPO-34
Framework type code	-**HOS**	**FAU**	**ETV**	**RTH**	**MFI**	**POR**	**CHA**
Reported space group	*Cmmm*	*Fd* 3 *m*	*P* 1	*C*2/*m*	*Pnma*	*P*42_1_*c*	*R* 3 *m*
*XDS*-identified space group	*C*222	*F*432	*P*1	*C*2	*P*222	*P*422	*R* 3
*SHELXT*-identified space group	*Cmmm*	*Fd* 3 *m*	*P* 1	*Cm*	*Pnma*	*P*42_1_*c*	*R* 3
*a* (Å)	14.65 (6)	25.08 (15)	9.04 (2)	10.10 (1)	13.58 (4)	15.08 (1)	14.01 (2)
*b* (Å)	51.87 (12)	25.08 (15)	9.77 (5)	21.90 (1)	19.82 (7)	15.08 (1)	14.01 (2)
*c* (Å)	13.10 (8)	25.08 (15)	10.81 (1)	10.48	20.22 (7)	19.22 (5)	15.34 (1)
α (°)	90	90	104.37 (8)	90	90	90	90
β (°)	90	90	98.32 (19)	96.02 (1)	90	90	90
γ (°)	90	90	99.49 (4)	90	90	90	120
Composition	Si_136_O_276_	Si_192_O_384_	Si_14_O_28_	Si_32_O_64_	Si_96_O_192_	P_32_Al_32_O_128_	P_18_Al_18_O_72_
No. of atoms in asymmetric unit	34	7	21	25	38	24	6
Rotation range (°)	91.09	92.20	110.17	80.67	105.28	94.78	53.76
Running time (min)	0.92	0.55	0.67	0.58	0.50	0.53	0.30
Completeness (%)	89.4	99.2	55.8	47.7	87.2	94.7	60.3
No. of unique reflections	5077	820	2033	1149	5639	2489	716
Reflection-to-parameter ratio	42.7	51.2	23.9	13.5	37.8	47.0	28.6
*I*/σ(*I*)	3.64	6.30	7.11	2.63	2.57	4.47	6.24
Resolution (Å)	0.80	0.80	0.80	0.80	0.80	0.80	0.80
*R*_int_ (%)	12.9	24.7	6.1	27.7	14.2	17.8	8.0
Initial *R*1 (%, all data)†	30.8	25.9	35.7	25.7	30.0	33.3	28.2

† The *R*1 values were calculated from the initial structure models given by *SHELXT*, prior to refinement.

**Table 2:** Summary of the crystallographic data and automated structure solution details of CAU-36 and pharmaceuticals The chemical compositions of CAU-36, acetamino­phen and biotin are difficult for *SHELXT* to assign correctly.

Sample	CAU-36	Acetamino­phen	Biotin
Reported space group	*P*4*c*2	*P*21*/n*	*P*2_1_2_1_2_1_
Identified space group (*XDS*)	*C*222	*P*2	*P*222
Identified space group (*SHELXT*)	*P*4*c*2	*P*21*/n*	*P*2_1_2_1_2_1_
*a* (Å)	21.95 (2)	7.11 (2)	5.30 (3)
*b* (Å)	21.95 (2)	9.38 (2)	10.47 (5)
*c* (Å)	8.74 (5)	11.72 (5)	21.34 (1)
α (°)	90	90.0	90.0
β (°)	90	97.19 (13)	90.0
γ (°)	90	90.0	90.0
Composition	C_232_N_80_O_16_P_12_	C_32_N_4_O_8_	C_40_N_8_O_12_S_4_
No. of atoms in the asymmetric unit	23	11	16
Running time (min)	0.40	0.38	0.28
Completeness (%)	94.9	91.1	89.5
No. of unique reflections	1886	1520	1307
Reflection-to-parameter ratio	21.4	33.8	20.1
*I*/σ(*I*)	4.23	3.05	3.56
Resolution (Å)	0.80	0.80	0.80
*R*_int_ (%)	24.6	14.3	13.8
Initial *R*1 (%)†	28.5	42.3	28.1

† The *R*1 values were calculated from the initial structure models given by *SHELXT*, prior to refinement.

**Table 3:** Summary of the crystallographic data and automated structure solution details for samples studied using different TEM platforms The chemical compositions of SCM-34 and AVAAGA peptide are difficult for *SHELXT* to assign correctly.

Sample	9,10-bis-[(perchloro-phenyl)-ethynyl]-anthracene (PPEA)	SCM-34	AVAAGA peptide
Detector	Ceta D	OneView	TemCam XF416
Microscope	Glacios Cryo-TEM	FEI Themis Z	Tecnai F30
Reported space group	*P*2_1_/*c*	*P* 1	*P*2_1_2_1_2_1_
Identified space group (*XDS*)	*P*2	*P*1	*P*2
Identified space group (*SHELXT*)	*P*2_1_/*c*	*P* 1	*P*2_1_
*a* (Å)	3.81 (1)	7.01 (2)	11.30 (3)
*b* (Å)	12.74 (1)	8.67 (1)	4.70 (5)
*c* (Å)	26.46 (1)	12.47 (1)	38.86 (10)
α (°)	90.0	101.91 (27)	90.0
β (°)	90.70 (10)	102.36 (14)	90.74 (10)
γ (°)	90.0	90.43 (24)	90.0
Composition	C_15_Cl_5_	C_14_N_2_Al_3_O_18_P_5_	C_35_O_13_N_8_
No. of atoms in asymmetric unit	20	22	56
Running time (min)	1.40	0.93	1.58
Completeness (%)	68.1	51.1	47.5
No. of unique reflections	1878	1513	2249
Reflection-to-parameter ratio	23.2	18.2	10.0
*I*/σ(*I*)	4.29	8.32	4.55
Resolution (Å)	0.80	0.80	0.80
*R*_int_ (%)	10.6	5.1	13.3
Initial *R*1 (%)†	25.7	27.9	32.5

† The *R*1 values were calculated from the initial structure models given by *SHELXT*, prior to refinement.
