# Supplementary material for: Automated and real-time structure solution using 3D electron diffraction
Source: J Appl Crystallogr. 2025 Oct 24;58(Pt 6):1986–94. doi: 10.1107/S1600576725008404 (PMC12810508; doi:10.1107/S1600576725008404)
Supplement: Supplementary file 5 [file j-58-01986-sup1.pdf]

JOURNAL OF APPLIED CRYSTALLOGRAPHY

**Volume 58 (2025)**

**Supporting information for article:**

# Automated and real-time structure solution using 3D electron diffraction

**Yi Luo, Yuwei Deng, Bin Wang, Junshu Chen, Weimin Yang and Xiaodong Zou**

## Methods

### Sample preparation for TEM

Continuous rotation electron diffraction (cRED) data were collected from the following materials: SCM-25 (Luo, Fu *et al.*, 2022), zeolite Y, EMM-37 (Kapaca *et al.*, 2019), RUB-13 (Luo, Wang *et al.*, 2023), ZSM-5, PST-14 (Seo, Yang *et al.*, 2018), AlPO-34, CAU-36 (Wang *et al.*, 2018), SCM-34 (Luo, Clabbers *et al.*, 2022), acetaminophen, and biotin. Zeolite Y, ZSM-5 and AlPO-34 were purchased from Shanghai Macklin Biochemical Co., Ltd. Acetaminophen and biotin were purchased from Shanghai Aladdin Biochemical Technology Co., Ltd. All samples were prepared using a standardized protocol: the powdered sample was initially crushed in an agate mortar, then suspended in ethanol (99.5 wt%) and dispersed by ultrasonication for 5 minutes to achieve homogeneous suspension. A drop of the resulting suspension was transferred onto a TEM grid with carbon film (CF200-Cu, 200 mesh, Electron Microscopy Sciences). After complete solvent evaporation at ambient conditions, the prepared grid was loaded into a normal single-tilt specimen holder for the cRED data collection at room temperature.

### Fully automated, real-time structure solution pipeline: *Instamatic-solve*

The 3D ED data for SCM-25 **(-HOS)**, zeolite Y **(FAU)**, EMM-37 **(ETV)**, RUB-13 **(RTH)**, ZSM-5 **(MFI)**, PST-14 **(POR)**, AlPO-34 **(CHA)**, CAU-36, Acetaminophen and Biotin were all acquired via the cRED method implemented in *Instamatic* (Smeets, 2018). This software was installed on a JEOL JEM2100 TEM (LaB6 filament, ASI Timepix camera) operating at 200 kV. The 3D ED dataset for SCM-34 was recorded using the cRED method implemented in *InsteaDMatic* (Roslova *et al.*, 2021) on a Themis Z FEI TEM (Gatan oneview camera) operating at 300 kV. Additionally, the 3D ED data of PPEA (Gorelik *et al.*, 2023) (https://doi.org/10.5281/zenodo.7322800) and AVVAGA (Gallagher-Jones *et al.*, 2020) (https://doi.org/10.5281/zenodo.5752657) were obtained from the Zenodo open-access repository.

*Instamatic-solve* (https://github.com/Junschen1/instamatic) is implemented in *Instamatic* software package (https://github.com/instamatic-dev/instamatic), with the overall process outlined in Figure 2. Briefly, once a 3D ED dataset is collected, its file path is immediately and automatically passed to a separate Python process, referred to as the "structure solution server". The server handles two main tasks: data reduction (via *XDS*) and structure solution (via *SHELXT*). In our lab, the microscope computer runs on Windows 7 while *XDS* requires a Linux environment. We therefore employ a VirtualBox Ubuntu virtual machine, communicated through the VirtualBox python API (https://pypi.org/project/pyvbox/). On Windows 10 or 11 systems, *XDS* can run using Windows subsystem for Linux 2 (WSL2), eliminating the need for VirtualBox. During data reduction, *XDS* uses empirical default parameters, and

we have incorporated an automatic resolution-cut function in the final data conversion step (*XDSCONV*). The default resolution range is defined from 20 to 0.8 Å in the *XDS.inp* file. Therefore, all datasets will initially be processed with a cutoff at 0.8 Å. Based on the statistics for this resolution range shown in *CORRECT.LP*, *Instamatic-solve* will further adjust the resolution cutoff when generating the *SHELX.hkl* intensity files using *XDSCONV.INP*. If reflections at 0.8 Å do not meet the criteria of $I/\sigma(I) \geq 0.3$, $CC_{1/2}$ is larger than or equal to 0.5 and flagged with a star(recommended by *XDS*), the cutoff will be automatically reduced to the highest resolution that satisfies these criteria. In this way, we avoid including too much noisy or useless high-resolution data, which may confuse the structure solution by *SHELXT*. After data reduction, *Instamatic* automatically generates a *SHELXT* input file, populating it with user-specified or *XDS*-derived information (space group and unit cell parameters). *SHELXT* is then invoked for structure solution, and the user is informed once any possible solutions are identified. The structure solution server can also operate in an offline mode by receiving a data path from the *Instamatic* GUI (Figure S1).

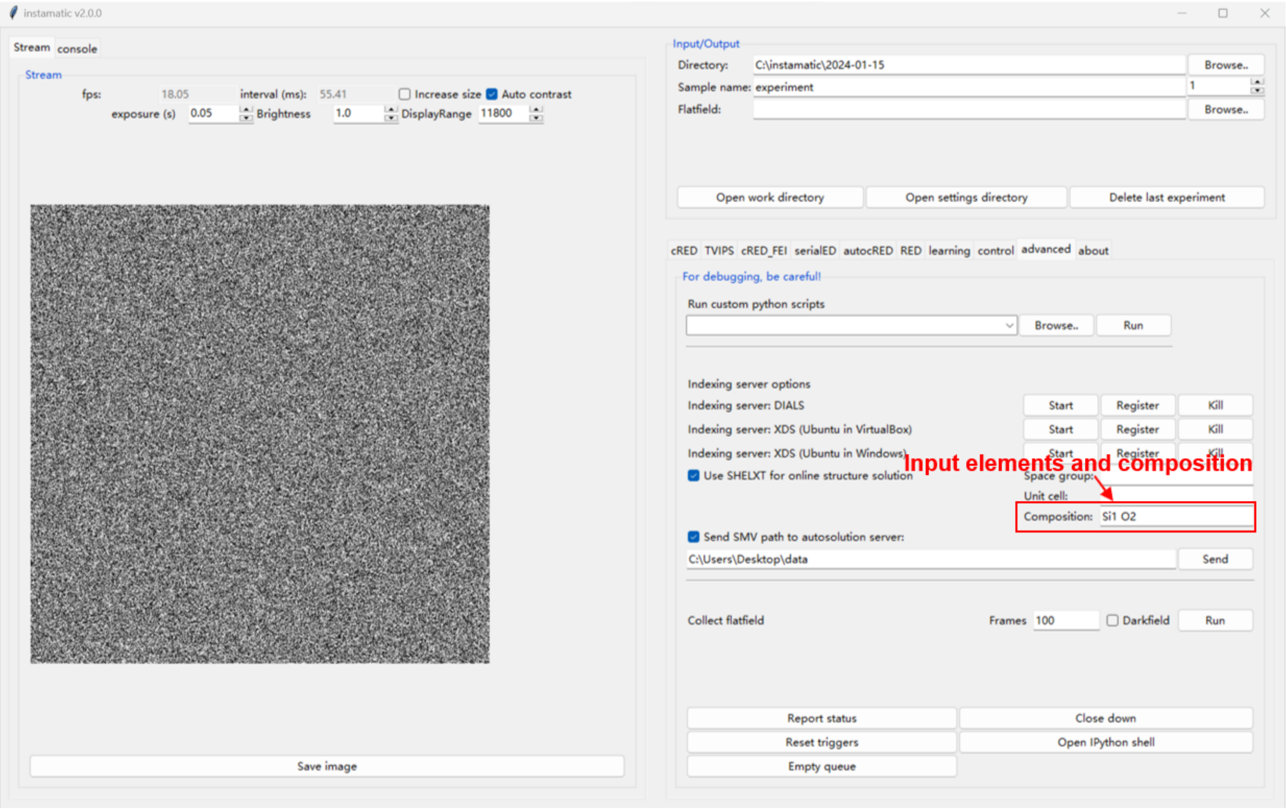


**Figure S1** The GUI of *Instamatic* with a preliminary element composition provided for *Instamatic-solve*.

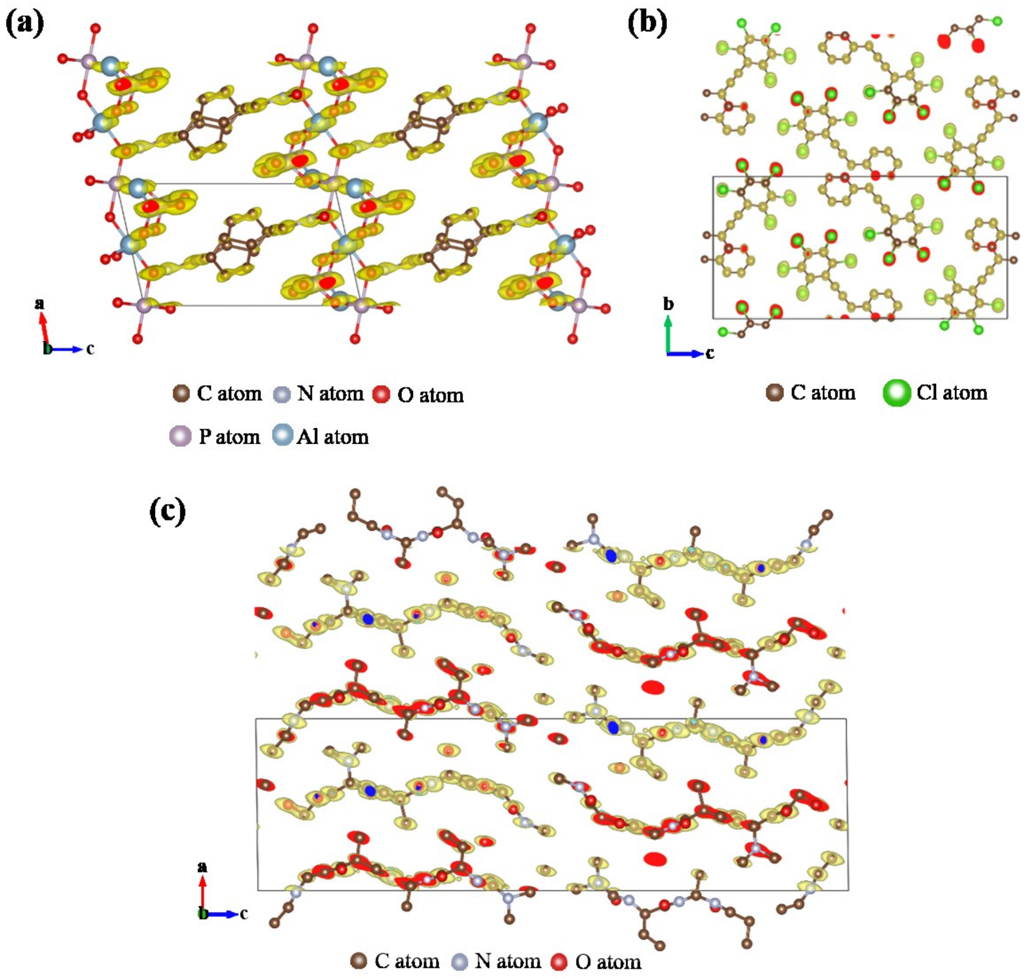


**Figure S2** Observed electrostatic potential maps and with the corresponding initial structures superimposed of (a) SCM-34, (b) PPEA and (c) AVAAGA.

**Table S1** Influence of data resolution on the automated structure solution results. Those giving successful structure solutions are highlighted in blue.

| Resolution (Å)[a] | | 0.8 | 0.9 | 1.0 | 1.1 | 1.2 | 1.3 | 1.4 | 1.5 |
|---|---|---|---|---|---|---|---|---|---|
| Completeness | **ETV** | 55.6[b] | 55.6 | 54.7 | 55.7 | 55.6 | 55.1 | 53.7 | 55.4 |
| | **RTH** | 47.7 | 47.9 | 47.6 | 48.8 | 49.1 | 49.1 | 49.7 | 49.2 |
| | **MFI** | 87.2 | 87.7 | 87.1 | 86.0 | 87.0 | 85.5 | 86.1 | 86.3 |
| | **FAU** | 99.2 | 99.5 | 99.2 | 98.9 | 100.0 | 99.6 | 100.0 | 100.0 |
| | **POR** | 94.7 | 94.8 | 94.1 | 93.6 | 93.4 | 93.4 | 92.2 | 91.4 |
| | **CHA** | 60.3 | 60.3 | 61.7 | 61.7 | 62.1 | 61.4 | 59.9 | 60.7 |
| | Acetaminophen | 91.1 | 89.6 | 87.7 | 88.1 | 88.3 | 88.5 | 87.1 | 86.4 |
| | Biotin | 89.5 | 90.2 | 89.7 | 90.4 | 89.5 | 89.8 | 90.3 | 89.0 |
| $N_{reflections}/N_{parameters}$[b] | **ETV** | 23.9 | 16.6 | 12.0 | 9.1 | 7.0 | 5.4 | 4.2 | 3.5 |
| | **RTH** | 13.5 | 9.5 | 5.4 | 3.9 | 3.2 | 2.5 | 2.1 | 1.6 |
| | **MFI** | 37.8 | 26.9 | 19.8 | 14.9 | 11.6 | 9.1 | 7.4 | 5.4 |
| | **FAU** | 51.2 | 40.4 | 30.3 | 23.5 | 18.6 | 15.0 | 12.2 | 10.2 |
| | **POR** | 47.0 | 33.4 | 24.7 | 18.9 | 14.7 | 11.7 | 9.5 | 7.8 |
| | **CHA** | 28.6 | 20.6 | 14.9 | 11.2 | 8.5 | 6.6 | 5.2 | 4.4 |
| | Acetaminophen | 33.8 | 23.6 | 17.0 | 13.0 | 9.9 | 7.9 | 6.3 | 5.1 |
| | Biotin | 20.1 | 14.6 | 10.9 | 8.4 | 6.5 | 5.1 | 4.3 | 3.5 |
| $I/\sigma(I)$ | **ETV** | 7.11 | 7.69 | 8.27 | 8.45 | 8.53 | 8.52 | 11.2 | 8.21 |
| | **RTH** | 2.63 | 2.85 | 3.12 | 3.36 | 3.53 | 3.47 | 3.35 | 3.39 |
| | **MFI** | 2.57 | 3.42 | 3.96 | 4.67 | 5.11 | 5.45 | 5.23 | 6.12 |
| | **FAU** | 6.30 | 6.53 | 6.61 | 7.18 | 7.46 | 6.83 | 7.07 | 7.08 |
| | **POR** | 4.47 | 5.11 | 5.49 | 5.87 | 6.43 | 6.70 | 6.40 | 6.17 |
| | **CHA** | 6.24 | 6.65 | 7.89 | 7.31 | 7.35 | 9.31 | 9.14 | 10.1 |
| | Acetaminophen | 3.05 | 3.79 | 4.47 | 4.79 | 5.44 | 5.52 | 6.19 | 6.37 |
| | Biotin | 3.56 | 4.30 | 4.55 | 4.95 | 5.04 | 5.28 | 5.50 | 5.06 |

[a] The dataset was cut at different resolutions to illustrate the requirement of the data quality for successful automated structure solution.

[b] $N_{reflections}$ represents the number of unique reflections, and $N_{parameters}$ denotes the number of parameters required to determine the framework structure during the automated structure solution (prior to refinement). The number of parameters refers to atomic coordinates (x, y, z) and isotropic displacement parameters ($U_{iso}$) that need to be determined in structure solution. Parameters for atoms located at special positions, where certain coordinate values are fixed by symmetry (e.g., 0.25, 0.5, 1.0, etc.), were excluded from the count. This ratio is used to evaluate the reliability and data redundancy of the structure solution. A higher ratio indicates that fewer parameters are being constrained by a larger amount of experimental data, typically increasing the reliability of the structure solution results.

**Table S2** Influence of data completeness on the automated structure solution. Different completeness cutoffs of 3D ED datasets were achieved by limiting the number of frames. Those giving successful structure solutions are highlighted in blue.

| Resolution (Å) | | 0.8 | | | | | | | |
|---|---|---|---|---|---|---|---|---|---|
| Completeness (%)[a] | | 100 | 90 | 80 | 70 | 60 | 50 | 40 | 30 |
| $N_{reflections}/N_{parameters}$[b] | **ETV** | -- | -- | -- | -- | -- | 21.5 | 17.1 | 12.8 |
| | **RTH** | -- | -- | -- | -- | -- | -- | 7.4 | 6.3 |
| | **MFI** | -- | 36.9 | 34.6 | 30.1 | 24.5 | 21.0 | 16.8 | 12.7 |
| | **FAU** | 51.2 | 51.1 | 45.4 | 39.8 | 33.7 | 28.1 | 22.4 | 16.3 |
| | **POR** | -- | 46.2 | 40.9 | 36.0 | 30.5 | 25.6 | 20.3 | 15.1 |
| | **CHA** | -- | -- | -- | -- | 28.5 | 23.6 | 17.8 | 12.4 |
| | Acetaminophen | -- | 33.4 | 29.7 | 25.8 | 22.5 | 18.1 | 14.6 | 11.4 |
| | Biotin | -- | 20.1 | 18.0 | 15.7 | 13.4 | 11.3 | 9.1 | 6.5 |
| $I/\sigma(I)$ | **ETV** | -- | -- | -- | -- | -- | 6.79 | 7.28 | 6.93 |
| | **RTH** | -- | -- | -- | -- | -- | -- | 4.19 | 4.21 |
| | **MFI** | -- | 2.43 | 2.37 | 2.54 | 2.61 | 2.37 | 1.74 | 2.47 |
| | **FAU** | 6.30 | 4.42 | 4.48 | 3.80 | 4.71 | 1.86 | 1.26 | 3.08 |
| | **POR** | -- | 4.43 | 4.23 | 4.63 | 4.42 | 4.41 | 3.91 | 3.09 |
| | **CHA** | -- | -- | -- | -- | 6.24 | 6.10 | 6.15 | 6.20 |
| | Acetaminophen | -- | 3.31 | 4.12 | 4.25 | 4.75 | 3.77 | 2.87 | 2.54 |
| | Biotin | -- | 3.56 | 4.09 | 4.25 | 3.94 | 4.12 | 3.87 | 2.91 |

[a] The dataset was cut at different completeness to illustrate the requirement of the data quality for successful automated structure solution. [b] $N_{reflections}$ denotes the number of unique reflections, while $N_{parameters}$ corresponds to the number of parameters required to determine the framework structure during the automated structure solution (prior to refinement).

## Supplementary notes

### 1.1 *Instamatic* Offline Setup Instructions (Windows Only)

**Operating system requirements**

*Instamatic* requires Windows 7 or higher.

#### 1.1.1 Install miniconda and create a conda virtual environment

Miniconda can be downloaded from the official website: https://www.anaconda.com/docs/getting-started/miniconda/install

To ensure compatibility, please use Conda v20.x.x or higher.

Create a new virtual environment using the following commands:

```
conda create -n instamatic python=3.7
conda activate instamatic
```

#### 1.1.2 Install *Instamatic* via pip

With the conda virtual environment activated, you can install *Instamatic* using the following command (install *git* if needed, available at https://gitforwindows.org/):

```
pip install git+https://github.com/Junschen1/instamatic.git
```

#### 1.1.3 Run *Instamatic*

After installation, you can run *Instamatic* as follows:

```
conda activate instamatic
instamatic
```

**Run *XDS* via Windows WSL2**

To run *XDS* on Windows using WSL2, ensure that Windows Subsystem for Linux 2 (WSL2) is installed first. If not, refer to the official installation guide:

https://learn.microsoft.com/en-us/windows/wsl/install

Then ensure that *XDS* is installed in WSL2. If not, refer to the official installation guide:

https://wiki.uni-konstanz.de/xds/index.php/Installation

Please follow the Linux guide and install the *XDS* package in WSL2.

Finally, ensure that *SHELXT* is installed in WSL2. If not, refer to the official installation guide:

https://shelx.uni-goettingen.de/

Once all dependencies are ready, you need to modify the configuration item in the *settings.yaml* file (If the user has

previously installed another version of Instamatic, the existing *setting.yaml* file should be replaced with the updated version available at: https://github.com/Junschen1/instamatic/tree/main/src/instamatic/config). This file is usually located in C:\Users****\AppData\Roaming\instamatic\config. However, if you can't find the file address, you can open *Instamatic* and look for the config directory information that pops up. The path shown there will lead you to the location of the *settings.yaml* file. Once you've located the file, change the name of the configuration item following "Win_XDS_PATH:" in the *settings.yaml* file to the directory where *XDS* was installed.

In the end, you can use the indexing server "XDS (Ubuntu in Windows)" in the advanced section of Instamatic. Note that the SMV file path sent to the autosolution server must not be located in your Ubuntu subsystem.

### 1.2 Instructions for the offline utilization of *Instamatic-solve*

For offline use, *Instamatic-solve* can be run on any computer running Windows 7 or later, provided the 3D ED data (in SMV, CBF, RAXIS, TIFF, or other XDS-compatible formats) are available.

#### 1.2.1 Update *XDS.inp* file

For data collected using the cRED method implemented in *Instamatic*, the *XDS.inp* (with all necessary experimental parameters automatically recorded) and SMV files are automatically generated without any human intervention. For the existing 3D ED datasets, if the *XDS.inp* is not provided, users can modify the fixed template by adjusting key parameters as follows (more details can be found via the link: https://xds.mr.mpg.de/html_doc/xds_parameters.html):

The essential parameters that must be modified in *XDS.inp* include (highlighted in red boxes in the figure):

- **DATA_RANGE** – Numbers of first and last data image collected.
- **SPOT_RANGE** – Numbers of the first and last data image used for locating strong spots.
- **BACKGROUND_RANGE** – Frame range for background estimation
- **STARTING_ANGLE** – Initial rotation angle
- **NX, NY** – Number of "fast" and "slow" pixels
- **QX, QY** – Size of "fast" and "slow" pixels (mm) along X and Y, respectively.
- **ORGX, ORGY** – The location of the direct beam on the frame (pixels)
- **DETECTOR_DISTANCE** – Sample-to-detector distance (mm)
- **OSCILLATION_RANGE** – Rotation step-size (angle)
- **ROTATION_AXIS** – Axis of rotation

The optional parameters for modification in *XDS.inp* (marked with blue boxes in the figure):

- **SPACE_GROUP_NUMBER** and **UNIT_CELL_CONSTANTS** – Required only if known; otherwise, *XDS* will determine them automatically.
- **INCLUDE_RESOLUTION_RANGE** – Default value: 20-0.8 Å (adjustable based on data quality).

```
! XDS.INP file for Rotation Electron Diffraction generated by `instamatic`
! Fri May 14 20:17:25 2021
! For definitions of input parameters, see:
! http://xds.mpimf-heidelberg.mpg.de/html_doc/xds_parameters.html
!
! cRED implementation reference paper:

! ********** Job control **********

!JOB= XYCORR INIT COLSPOT IDXREF
!JOB= DEFPIX INTEGRATE CORRECT
!JOB= CORRECT

MAXIMUM_NUMBER_OF_JOBS=4
MAXIMUM_NUMBER_OF_PROCESSORS=4

! ********** Data images **********

NAME_TEMPLATE_OF_DATA_FRAMES= data/frame_???.img   SMV
DATA_RANGE=              1 118
SPOT_RANGE=              1 118
BACKGROUND_RANGE=        1 118

! ********** Crystal **********

!SPACE_GROUP_NUMBER= 0
!UNIT_CELL_CONSTANTS= 10 20 30 90 90 90

!REIDX=                       !Optional reindexing transformation to apply on reflection indices
FRIEDEL'S_LAW=TRUE            !TRUE is default

!phi(i) = STARTING_ANGLE + OSCILLATION_RANGE * (i - STARTING_FRAME)
STARTING_ANGLE= -60.0
STARTING_FRAME= 1

MAX_CELL_AXIS_ERROR=  0.05      !0.03 is default
MAX_CELL_ANGLE_ERROR= 3.0       !2.0  is default

TEST_RESOLUTION_RANGE=10.0 1.0 !for calculation of Rmeas when analysing the intensity data for space group symmetry in the CORRECT step.
!MIN_RFL_Rmeas=50             !50 is default - used in the CORRECT step for identification of possible space groups.
!MAX_FAC_Rmeas=2.0            !2.0 is default - used in the CORRECT step for identification of possible space groups.

! ********** Detector hardware **********

NX=2048     NY=2048            !Number of pixels
QX=0.0280  QY=0.0280          !Physical size of pixels (mm)
OVERLOAD= 130000              !default value dependent on the detector used
TRUSTED_REGION= 0.0  1.3  !default "0.0 1.05". Corners for square detector max "0.0 1.4142"
!UNTRUSTED_RECTANGLE= 255 262 0 517
!DETECTOR= PILATUS      ! Pretend to be PILATUS detector to enable geometric corrections
!X-GEO_CORR= XCORR.cbf  ! X stretch correction
!Y-GEO_CORR= YCORR.cbf  ! Y stretch correction

SENSOR_THICKNESS=0.30
AIR=0.0

! ********** Trusted detector region **********

VALUE_RANGE_FOR_TRUSTED_DETECTOR_PIXELS= 10 300000 !Values are defined in `ABS.CBF`, check mask in `BKGPIX.CBF`, used in DEFPIX
!MINIMUM_ZETA=                                      !0.05 is default

INCLUDE_RESOLUTION_RANGE= 20 0.80

!Ice Ring exclusion, important for data collected using cryo holders
!EXCLUDE_RESOLUTION_RANGE= 3.93 3.87       !ice-ring at 3.897 Angstrom
!EXCLUDE_RESOLUTION_RANGE= 3.70 3.64       !ice-ring at 3.669 Angstrom
!EXCLUDE_RESOLUTION_RANGE= 3.47 3.41       !ice-ring at 3.441 Angstrom (Main)
!EXCLUDE_RESOLUTION_RANGE= 2.70 2.64       !ice-ring at 2.671 Angstrom
!EXCLUDE_RESOLUTION_RANGE= 2.28 2.22       !ice-ring at 2.249 Angstrom (Main)
!EXCLUDE_RESOLUTION_RANGE= 2.102 2.042     !ice-ring at 2.072 Angstrom - strong
!EXCLUDE_RESOLUTION_RANGE= 1.978 1.918     !ice-ring at 1.948 Angstrom - weak
!EXCLUDE_RESOLUTION_RANGE= 1.948 1.888     !ice-ring at 1.918 Angstrom - strong
!EXCLUDE_RESOLUTION_RANGE= 1.913 1.853     !ice-ring at 1.883 Angstrom - weak
!EXCLUDE_RESOLUTION_RANGE= 1.751 1.691     !ice-ring at 1.721 Angstrom - weak

! ********** Detector geometry & Rotation axis **********

DIRECTION_OF_DETECTOR_X-AXIS= 1 0 0
DIRECTION_OF_DETECTOR_Y-AXIS= 0 1 0

ORGX= 1010    ORGY= 1049         !Detector origin (pixels). Often close to the image center, i.e. ORGX=NX/2; ORGY=NY/2
DETECTOR_DISTANCE= +1139.67            !Can be negative. Positive because the detector normal points away from the crystal.

OSCILLATION_RANGE= 1
!OSCILLATION_RANGE 0.2306              !Calibrated value if above one is too far off

ROTATION_AXIS= -0.998 -0.0627 0.0000

! ********** Incident beam **********

X-RAY_WAVELENGTH= 0.0251              !used by IDXREF
INCIDENT_BEAM_DIRECTION= 0 0 1        !The vector points from the source towards the crystal

! ********** Background and peak pixels **********

!NBX=5  NBY=5            ! 3 is default, used to estimate the expected variation in a data image, see GAIN.cbf
!BACKGROUND_PIXEL= 6.0   ! Background pixel belongs to background if variation less than given esds
!STRONG_PIXEL= 3.0       ! Strong pixel must exceed background by more than number of given esds
!MAXIMUM_NUMBER_OF_STRONG_PIXELS= 1500000 ! Approximate upper limit for the total number of 'strong' pixels
!MINIMUM_NUMBER_OF_PIXELS_IN_A_SPOT= 6    ! Used to suppress spurious, isolated 'strong' pixels from entering the spot list
!SPOT_MAXIMUM-CENTROID= 3.0               ! Maximum deviation of spot maximum from spot centroid
!SIGNAL_PIXEL= 3.0                       ! Signal pixels must exceed background by more than given esds

! ********** Refinement **********

 REFINE(IDXREF)=    BEAM AXIS ORIENTATION CELL !POSITION
 REFINE(INTEGRATE)= !POSITION BEAM AXIS !ORIENTATION CELL
 REFINE(CORRECT)=   BEAM AXIS ORIENTATION CELL !POSITION

! ********** Indexing **********

MINIMUM_FRACTION_OF_INDEXED_SPOTS= 0.25   !0.50 is default.
!MAXIMUM_ERROR_OF_SPOT_POSITION=6.0       ! 3.0 is default
DELPHI= 20
```

**Figure S3** The template of *XDS.inp* file is shown in this figure.

### 1.2.2 Launch *Instamatic-solve*

Once the *XDS.inp* file is ready, the offline automated structure solution will be proceeded as follows:

Launch *Instamatic* via Windows WSL2.

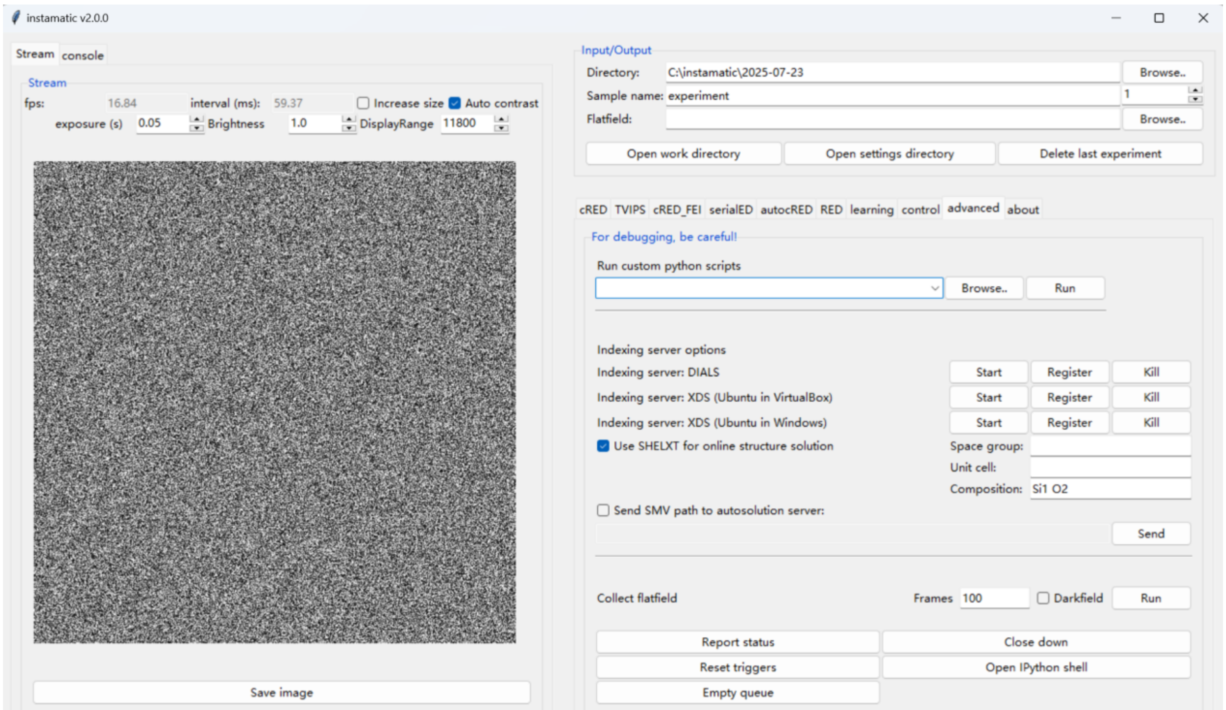


### 1.2.3 Input chemical composition

Start the *XDS* server and input an approximate chemical composition of the sample.

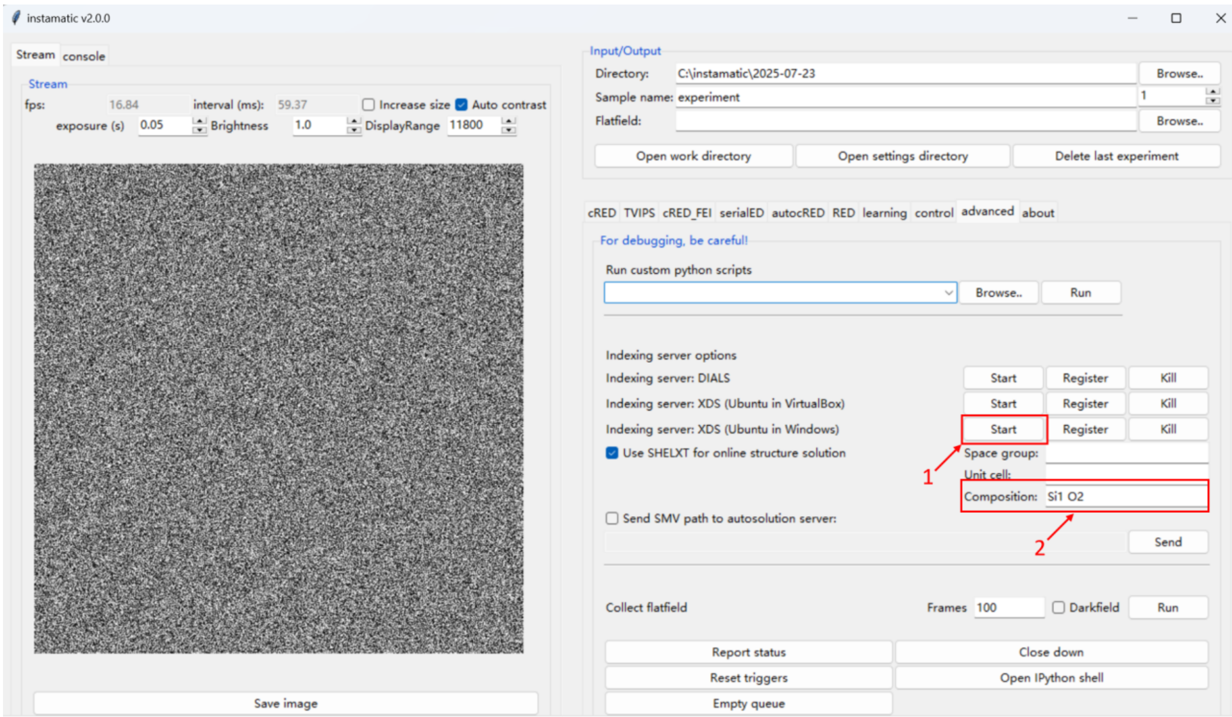

### 1.2.4 Input the path of the data folder

Provide the folder path containing the 3D ED data and *XDS.inp*.

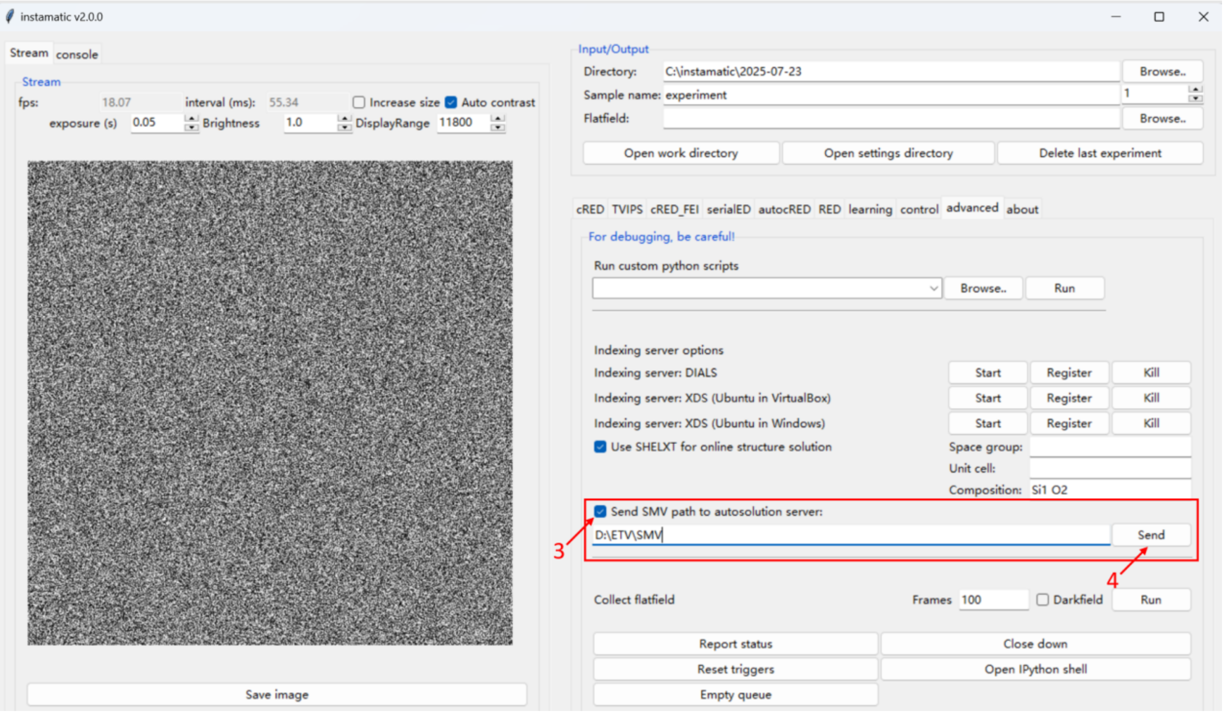


### 1.2.5 Run the pipeline

Send the path and run the offline automated structure solution.

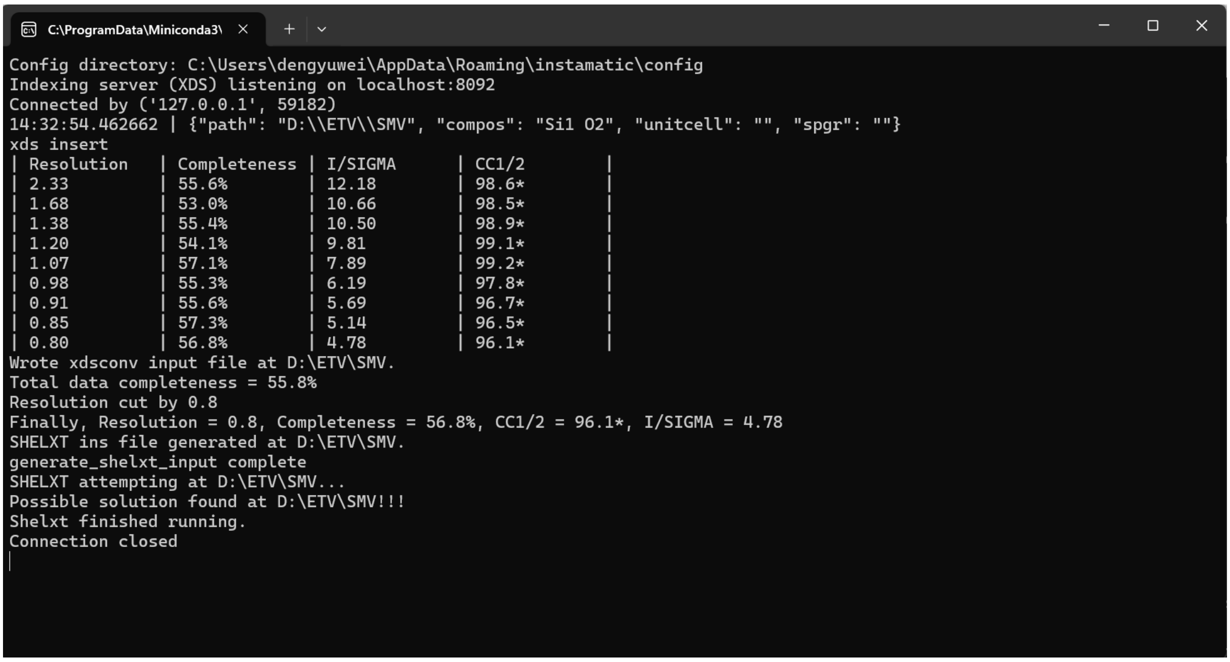

```
C:\ProgramData\Miniconda3\
Config directory: C:\Users\dengyuwei\AppData\Roaming\instamatic\config
Indexing server (XDS) listening on localhost:8092
Connected by ('127.0.0.1', 59182)
14:32:54.462662 | {"path": "D:\\ETV\\SMV", "compos": "Si1 O2", "unitcell": "", "spgr": ""}
xds insert
| Resolution   | Completeness | I/SIGMA     | CC1/2       |
| 2.33         | 55.6%        | 12.18       | 98.6*       |
| 1.68         | 53.0%        | 10.66       | 98.5*       |
| 1.38         | 55.4%        | 10.50       | 98.9*       |
| 1.20         | 54.1%        | 9.81        | 99.1*       |
| 1.07         | 57.1%        | 7.89        | 99.2*       |
| 0.98         | 55.3%        | 6.19        | 97.8*       |
| 0.91         | 55.6%        | 5.69        | 96.7*       |
| 0.85         | 57.3%        | 5.14        | 96.5*       |
| 0.80         | 56.8%        | 4.78        | 96.1*       |
Wrote xdsconv input file at D:\ETV\SMV.
Total data completeness = 55.8%
Resolution cut by 0.8
Finally, Resolution = 0.8, Completeness = 56.8%, CC1/2 = 96.1*, I/SIGMA = 4.78
SHELXT ins file generated at D:\ETV\SMV.
generate_shelxt_input complete
SHELXT attempting at D:\ETV\SMV...
Possible solution found at D:\ETV\SMV!!!
Shelxt finished running.
Connection closed
```

A demonstration of this workflow for **ETV** zeolite is also provided in Video 3.

## References


Andrew, P.& Diederichs, K. (2012). *Science* **336**, 1030-1033.

Gallagher-Jones, M., Bustillo, K. C., Ophus, C., Richards, L. S., Ciston, J., Lee, S., Minor, A. M. & Rodriguez, J. A. (2020). *IUCrJ* **7**, 490–499.

Kapaca, E., Burton, A., Terefenko, E., Vroman, H., Weston, S. C., Kochersperger, M., Afeworki, M., Paur, C., Koziol, L., Ravikovitch, P., Xu, H., Zou, X. & Willhammar, T. (2019). *Inorg. Chem.* **58**, 12854–12858.

Luo, Y., Clabbers, M. T. B., Qiao, J., Yuan, Z., Yang, W. & Zou, X. (2022). *J. Am. Chem. Soc.* **144**, 10817–10824.

Luo, Y., Fu, W., Wang, B., Yuan, Z., Sun, J., Zou, X. Yang, W. (2022). *Inorg. Chem.* **61**, 4371–4377.

Luo, Y., Wang, B., Smeets, S., Sun, J., Yang, W. & Zou, X. (2023). *Nat. Chem.* **15**, 483-490.

Roslova. M., Smeets. S., Wang. B., Thersleff. T., Xu. H. & Zou. X. (2020). *J. Appl. Cryst*. **53**, 1217-1224.

Seo, S., Yang, T., Shin, J., Jo, D., Zou, X. & Hong, S. (2018). *Angew. Chem. Int. Ed*. **57**, 1-7.

Smeets, S. (2018). *Instamatic, a Python Program to Collect Serial and Rotation Electron Diffraction Data,* https://github.com/stefsmeets/ instamatic.

Gorelik, T. E., Ulmer, A., Schleper, A L. & Kuehne, A. J. C. (2023). *Z. Kristallogr.* **238**, 253-260.

Wang, B., Rhauderwiek, T., Inge, A. K., Xu, H., Yang, T., Huang, Z., Stock, N. & Zou, X. (2018). *Chem. Eur. J.* **24**, 17429-17433.

Wang, L., Chen, Y., Emma, S. H., Pål, S., Gerhard, H., Xu, H & Zou, X. (2025). *bioRxiv*: 2025-04.
